# Distinct Progenitor Origin Distinguishes a Lineage of Dendritic-Like Cells in Spleen

**DOI:** 10.3389/fimmu.2013.00501

**Published:** 2014-01-02

**Authors:** Sawang Petvises, Helen Christine O’Neill

**Affiliations:** ^1^Stem Cell and Immunology Lab, Division of Biomedical Sciences, Research School of Biology, Australian National University, Canberra, ACT, Australia

**Keywords:** hematopoiesis, progenitor, dendritic cell, stroma

## Abstract

The dendritic cell (DC) compartment comprises subsets of cells with distinct phenotypes. Previously this lab reported methodology for hematopoiesis of dendritic-like cells *in vitro* dependent on a murine splenic stromal cell line (5G3). Co-cultures of lineage-depleted bone marrow (Lin^−^ BM) over 5G3 continuously produced a distinct population of dendritic-like “L-DC” for up to 35 days. Here the progenitor of L-DC is investigated in relation to known BM-derived hematopoietic progenitors. It is shown here that L-DC-like cells also derive from the CD150^+^Flt3^−^ long-term reconstituting-hematopoietic stem cells (HSC), and also from the Flt3^+^ multipotential progenitor subset in BM. Lin^−^ BM co-cultures also produce a transient population of cells resembling conventional (c) DC. Production of cDC-like cells is shown here to be transient and M-CSF dependent, and also appears following co-culture of described common dendritic progenitors or monocyte dendritic progenitors over 5G3. BM cells from C57BL/6-*flt3L^tm1lmx^* and C57BL/6-*Csf2^tm1Ard^* mice which lack cDC precursors and monocytes, are shown here to contain L-DC progenitors which can seed 5G3 co-cultures. L-DC are functionally distinct cells, in that they arise independently of M-CSF, and by direct differentiation from HSC.

## Introduction

Dendritic cells (DCs) are professional antigen presenting cells required for induction of adaptive immune responses against invading pathogens, and for maintaining tolerance to self antigens. DC recognize and take up foreign substances via a number of pathogen-specific receptors. Internalized native antigens are then processed into small peptides, and displayed in the context of major histocompatibility complex (MHC) Class I and Class II molecules for activation of CD8^+^ and CD4^+^ T cells, respectively (1, 2). Broadly, DC residing in lymphoid tissues can be categorized as tissue resident DC or conventional (c) DC, plasmacytoid (p) DC, migratory DC, and inflammatory DC (3). A complexity of DC subsets enables the host to generate a range of immune responses against harmful pathogens.

A distinct subset of dendritic-like cells, namely “L-DC,” was recently characterized in murine spleen in the steady-state and also produced in co-cultures of mouse spleen or bone marrow (BM) cells over stroma (4–6, 31). L-DC express CD11b and CD11c molecules on their cell surface, but not other DC markers such as MHC-II, CD8α, or B220. Cells have high capacity to endocytose antigen and to induce CD8 T cell activation (5, 8, 31). L-DC produced *in vitro* or isolated from spleen have been shown to have powerful capacity to cross-present antigen to CD8^+^ T cells (7, 9). They can be distinguished functionally and phenotypically from both DC and monocyte subsets in spleen (9). We have previously identified both BM and spleen as a source of hematopoietic progenitors which can seed splenic stroma for L-DC production (11–13, 31). Here the L-DC progenitor is investigated in detail in relation to hematopoietic stem/progenitor cell subsets described previously in BM. Development of L-DC from progenitors has also been characterized in terms of dependency for known cytokines which support the development of other known DC and myeloid subsets.

Most DC in lymphoid tissues have a short life span and are thought to be repopulated by committed progenitors arising in BM. Myeloid and dendritic progenitors (MDP) were recently described as c-kit^hi^Lin^−^Sca-1^−^Flt3^+^ cells which also express CD115 and CX3CR1 (14, 15). Myeloid progenitors (MP) were described as c-kit^hi^Lin^−^Sca-1^−^Flt3^+^ cells expressing CD115 but not CX3CR1 (14, 15) and a common dendritic progenitor (CDP) was identified in BM which produces both cDC and pDC (15, 16). The lineage relationship between L-DC with cDC/pDC was addressed by sorting purified BM progenitors and assessing their capacity to differentiate when co-cultured over the 5G3 stromal line to give cDC, pDC, L-DC, and monocyte/myeloid cells. These studies also tested the CD150^+^Flt3^−^ subset of long-term (LT) hematopoietic stem cells (HSC), and the CD150^−^Flt3^+^ subset of short-term (ST)-HSC from BM, also referred to as multipotential progenitors (MPP) (17–19).

Using splenic stromal co-cultures to induce differentiation of dendritic-like cells from progenitors in BM, we have distinguished the L-DC progenitor from known subsets of CDP, MDP, and MP, confirming a distinct lineage origin for these cells. The production of cDC-like cells in co-cultures is also described in terms of a transient cell population which is distinct from L-DC.

## Materials and Methods

### Animals

Specific pathogen-free female C57BL/6J mice were bred at the John Curtin School of Medical Research (JCSMR) (Canberra, ACT, Australia). B6.129P(Cg)-*Ptprc^a^* *Cx3cr1^tm1Litt^*/LittJ mice (CX3CR1-GFP) were purchased from Walter and Eliza Hall Institute (Melbourne, VIC, Australia) (20). C57BL/6-*flt3L^tm1lmx^* mice (Flt3L^−/−^) (Taconic Farms Inc., NY, USA) were purchased from the Biomedical Research Facility, University of Western Australia (Perth, WA, Australia) and C57BL/6-*Csf2^tm1Ard^* (GM-CSF^−/−^) mice (21) were obtained from the Ludwig Institute for Cancer Research (Melbourne, VIC, Australia). Animal housing, handling, and experimentation was approved by the Animal Experimentation Ethics Committee (Australian National University, Canberra, ACT, Australia). Animals were sacrificed by cervical dislocation.

### Antibodies

Fluorochrome-conjugated antibodies specific for CD11c (N418), CD11b (M1/70), CD115 (AFS98), and streptavidin-APC-Cy7 were obtained from eBioscience (San Diego, CA, USA) or BioLegend (San Gabriel, CA, USA). Fluorochrome-conjugated antibodies specific for CD8α (53-6.7), B220 (RA3-6B2), MHC-II (AF6-120.1), F4/80 (C1: A3-1), c-kit (2B8), Sca1 (E13-161.7), Flt3 (A2F10), CD43 (1B11), Sirpα (P84), CD45RB (C363.16A), CD150 (TC15-12F12.2), 4-1BBL (TKS-1), streptavidin-PE-Cy7, streptavidin-PE, and streptavidin-FITC were obtained from BioLegend (San Gabriel, CA, USA). Goat-anti rat-PE-Texas Red was obtained from Invitrogen (Eugene, OR, USA). Isotype control antibodies including Rat IgG_2a_-FITC (R35-95), Rat IgG_2b_-PE (RTK4530), Rat IgG_2b_-PE-Cy7 (eB149/10H5), Mouse IgG_2a_-biotin (eBM2a), and Hamster IgG-APC (eBio299Arm) were obtained from eBioscience.

### Cell culture and reagents

Cells were cultured in Dulbecco’s modified Eagles Medium (DMEM) supplemented with 4 g/L d-glucose, 6 mg/L folic acid, 36 mg/L l-asparagine, 116 mg/L l-arginine, to which was added 10% fetal calf serum (FCS), 10 mM HEPES, 2 mM l-glutamine, 100 U/L penicillin, 100 μg/L streptomycin, and 5 × 10^−5^ M 2-mercaptoethanol. The splenic stromal cell line 5G3 was passaged every 4 days by scraping and transferring non-adherent cells to a new flask (6). Cells were maintained in 5% CO_2_ in 95% humidity at 37°C.

### Preparation of BM cells

Bone marrow was isolated by flushing femurs and tibias with 5 mL of DMEM. Cells were centrifuged and resuspended in red blood cell lysis buffer [140 mM NH_4_CL, 17 mM Tris Base (pH 7.5)]. Lin^−^ BM was prepared using a lineage depletion kit (Miltenyi Biotech, Gladbach, Germany) and MACS^®^ magnetic bead technology (Miltenyi Biotech). The lineage depletion cocktail (Miltenyi Biotech) comprised biotinylated antibodies specific for all hematopoietic lineages (7–4, CD5, CD11b, B220, Ly6G/C, and Ter119). Antibody specific for CD11c (HL3: Becton Dickinson Pharmingen, San Diego, CA, USA) was also added to deplete DC. Anti-biotin microbeads in MS or LS columns (Miltenyi Biotech) were used to purify Lin^−^ cells.

### Co-cultures over 5G3 stroma

For establishment of co-cultures, Lin^−^ BM or sorted hematopoietic progenitors (10^4–5^ cells/mL) were overlaid on to near-confluent 5G3 stroma in replicate 25 cm^2^ flasks (5 mL). Medium change was performed every 3–4 days by replacement of 2.5 mL medium with 2.5 mL fresh, warmed complete medium. Non-adherent cells produced in co-cultures were collected at days 14, 21, 28 and 35 for analysis of cell subsets produced. For inhibition studies, 10^4–5^ cells/mL Lin^−^ BM from C57BL/6J mice were overlaid on to 5G3 stroma in the presence and absence of inhibitors. These were titrated before use to find optimal inhibitory concentrations. These included a M-CSFR inhibitor GW2580 used at 10 nM (BioVision, CA, USA), and a Flt3 inhibitor used at 0.5 μM (Catalog #343202: Merck KGa, Darmstadt, Germany). The Flt3 inhibitor is a cell-permeable thienylcarboxamide compound which acts as a potent ATP-competitive selective Flt3 inhibitor with little effect on other kinases. In each case non-adherent cells were collected at days 14, 21, and 28 for analysis of cells produced. For Flt3 inhibition experiments, cultures of Lin^−^ BM supplemented with 200 ng/mL Flt3L (eBioscience) were established as controls.

### Isolation of hematopoietic progenitors

Lin^−^ BM was prepared as described above and then stained with antibodies for delineation of progenitors for sorting. A cocktail of antibodies (Miltenyi Biotech) was used to sort out Lin^−^ BM in order to further gate undifferentiated cells. This cocktail recognized BM-derived cells expressing CD3ε, Thy1.2, CD19, NK1.1, Gr-1, CD11b, Ter119, MHC-II, or CD11c. LT-HSC were sorted as the CD150^+^Flt3^−^ subset of c-kit^+^Lin^−^Sca-1^+^ (KLS) cells (19); MPP as CD150^−^Flt3^+^ KLS cells (19); MDP as Lin^−^Sca-1^−^c-kit^hi^Flt3^+^CD115^+^CX3CR1^+^ cells (14, 15); and MP as Lin^−^Sca-1^−^c-kit^hi^Flt3^+^CD115^+^CX3CR1^−^ cells (14). In some experiments, MDP were sorted as Lin^−^Sca-1^−^c-kit^hi^Flt3^+^CD115^+^ cells (14) and CDP as Lin^−^Sca-1^−^c-kit^lo^Flt3^+^CD115^+^ cells (16, 22). Sorting was performed on a FACS Aria II flow cytometer (Becton Dickinson, Franklin Lakes, NJ, USA), and sorted cells reanalyzed to check purity. Isotype control antibodies were used to set gates and propidium iodide (PI: 1 μg/mL) staining of cells was used for dead cell discrimination. Sorted cells were washed twice and overlaid on to 5G3 stroma for assessment of cell production.

### Analysis of cells produced in co-cultures

Cells collected from co-cultures at each time point were stained with antibodies specific to dendritic and myeloid cells. Briefly, 10^5–6^ cells were firstly incubated with purified CD16/32 antibody (Clone 93; eBioscience) for 15 min to block surface Fc receptors. Cells were then washed with DMEM/1% FCS/0.1% NaN_3_ and stained with primary antibodies specific for CD11c, CD11b, CD8α, B220, and MHC-II for 20 min on ice. In some experiments, antibody to F4/80 and 4-1BBL were used to detect specific subsets in co-cultures. Any secondary reagents were added to the stained cells after a washing step, and further incubated for 20 min on ice. Cells were finally washed twice and resuspended in DMEM/1% FCS/0.1% NaN_3_ for flow cytometric analysis. Cells were stained with PI (1 μg/mL) for live cell discrimination. Cell acquisition was performed using a LSR II flow cytometer (Becton Dickinson). Between 5 × 10^4^ and 1 × 10^6^ events were collected for each sample. Gates were set to delineate cell subsets using isotype control antibodies and “fluorescence minus one” (FMO) controls. Cell subset analysis was performed using BD FACSDiva Software (Becton Dickinson) and FlowJo Software (Tristar; Phoenix, AZ, USA).

### Statistical analysis

In some figures replicated data are presented as mean ± SE (*n* = 3). The Wilcoxon Rank Sum Test was used to test significance (*p* < 0.05).

## Results

### L-DC and cDC-like cells arise from distinct progenitors in BM

**Figure 1:**
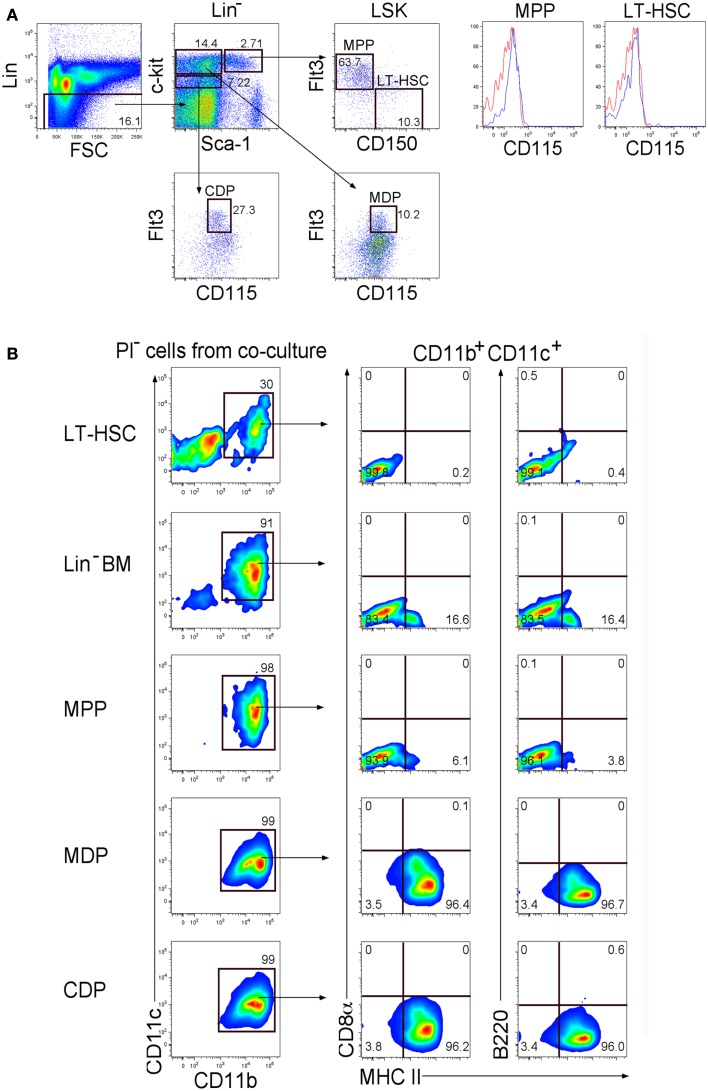
**Differentiative capacity of hematopoietic progenitors in stromal co-cultures**. **(A)** Lin^−^ BM from C5BL/6J mice was stained with antibodies to distinguish hematopoietic progenitors flow cytometrically. Cells were initially stained with an antibody cocktail specific for CD3ε, CD4, CD5, CD8α, B220, Gr-1, CD11b, Ter119, and CD11c to gate out Lin^+^ cells. Sca-1 and c-kit staining was used to identify Lin^−^c-kit^hi^Sca-1^+^(LSK), Lin^−^c-kit^hi^Sca-1^−^, and Lin^−^c-kit^lo^Sca-1^−^subsets. Staining with Flt3 and CD150 was used to distinguish LT-HSC and MPP. These two subsets were tested for expression of CD115 (red) in relation to isotype control (blue). CDP and MDP were distinguished further on the basis of Flt3 and CD115 expression. Propidium iodide (PI) staining was used to discriminate dead cells. Gates were set on bivariate plots using isotype control antibodies and numbers on gates reflect % positive cells. **(B)** Each of the progenitor subsets shown in **(A)** was sorted and overlaid in 5G3 stromal co-cultures, with Lin^−^BM as a control. Cell production was assessed over time using multicolor flow cytometry to delineate cell subsets amongst non-adherent cells collected at medium change. Data shows production of CD11b^+^CD11c^+^ cells gated amongst the PI^−^ live cell population at 21 days for one of three replicate cultures established for each progenitor type. Cells were also tested for CD8α, B220, and MHC-II staining as described in **(A)**.

Previous reports from this lab showed that splenic stroma can support longterm development of L-DC from Lin^−^ BM along with a transient population of cDC-like cells (6, 8). To identify progenitors of these distinct cell types, known hematopoietic progenitor subsets in BM were sorted and overlaid on to 5G3 stroma to determine their differentiative capacity. Sorting involved gating Lin^−^ cells and delineation of progenitors differing in c-kit and Sca-1 expression. Subsets were further divided on the basis of expression of Flt3, CD150, and CD115 to give LT-HSC, MPP, CDP, and MDP (Figure 1A). The MDP and CDP populations were distinct as Flt3^+^CD115^+^ cells differing in level of c-kit expression. LT-HSC and MPP differed in expression of CD150 and Flt3, and did not express CD115.

Co-cultures established with LT-HSC, MPP, CDP, MDP, and Lin^−^ BM (as control) were maintained in triplicate, and cell production analyzed weekly by staining non-adherent cells collected at medium change for expression of CD11c, CD11b, B220, CD8α, and MHC-II. Co-cultures produced only myeloid and dendritic cells (CD11b^+^, CD11c^+^) along with a progenitor-containing (CD11b^−^, CD11c^−^) subset but no lymphoid lineage cells identifiable by B220 or CD8α staining (Figure 1B). After 21 days, co-cultures of LT-HSC produced CD11b^+^CD11c^+^MHC-II^−^L-DC-like cells and a CD11b^−^CD11c^−^ progenitor-like subset, while co-cultures of CDP and MDP produced only CD11b^+^CD11c^+^MHC-II^+^ cDC-like cells. Co-cultures of Lin^−^BM produced progenitors, along with both dendritic-like subsets as reported previously (6–8). MPP produced a majority of L-DC-like cells with 6.1% cDC-like cells.

**Figure 2:**
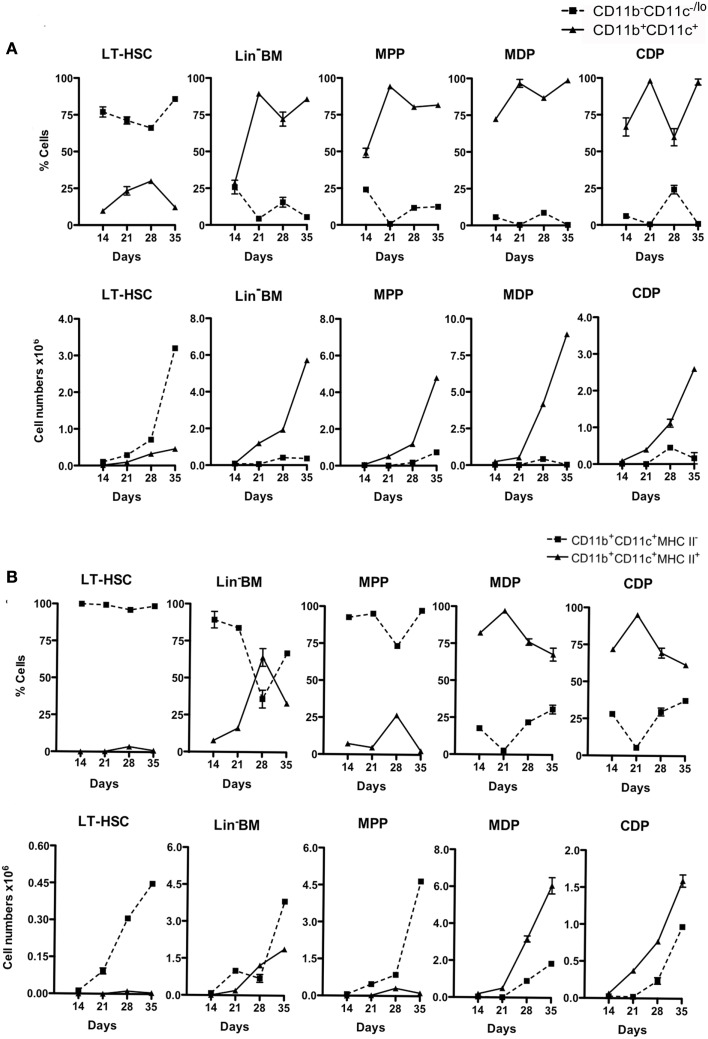
**Differential production of dendritic-like cells in stromal co-cultures established with different progenitors**. Co-cultures were established over 5G3 as in Figure 1. Cell production was assessed after 14, 21, 28, and 35 days using antibody staining and multicolor flow cytometry. Cell production was assessed in terms of proportion of each subset, and number of cells of each type produced. Graphs show mean ± SE for triplicate co-cultures. **(A)** Production of CD11b^−^CD11c^−/lo^ and CD11b^+^CD11c^+^ cells. **(B)** Production of CD11b^+^CD11c^+^ cells differing in expression of MHC-II. All data for days 21, 28, 35, but only some 14 days data, are significantly different (*p* ≤ 0.05).

Production of cells from triplicate co-cultures was monitored across 14, 21, 28, and 35 days. The reproducibility of the co-culture system and of subset analysis by flow cytometry is indicated by the low error obtained in assessing triplicate cultures. The relative proportion of progenitors (CD11b^−^CD11c^−/lo^) to mature (CD11b^+^CD11c^+^) cells differed across co-cultures (Figure 2A). LT-HSC co-cultures maintained >75% progenitors out to 35 days, with all other co-cultures maintaining ≤25%, reaching zero by 35 days for all but MPP co-cultures. The absolute number of cells produced also varied since progenitors varied in ability to seed differentiation. LT-HSC co-cultures were distinct, with lower overall production of CD11b^+^CD11c^+^ cells and a much higher production of CD11b^−^CD11c^−/lo^ progenitors (Figure 2A). Further analysis of mature CD11b^+^CD11c^+^ cells distinguished the MHC-II^−^ L-DC-like and the MHC-II^+^ cDC-like subsets. LT-HSC co-cultures produced only L-DC-like cells across 14–35 days (Figure 2B) with almost no cDC-like cells. Lin^−^ BM and MPP co-cultures produced both cell types. MPP co-cultures showed a clear majority of L-DC, while Lin^−^ BM co-cultures produced both cell types although the proportion of cDC-like cells declined after 21 days. Co-cultures of MDP and CDP produced a high proportion of cDC-like cells, with low L-DC production evident by 35 days (Figure 2B). This could relate to the presence of low numbers of contaminant L-DC progenitors amongst sorted CDP and MDP. Similar data have been obtained over many replicate experiments (all not shown), although the size of subsets has been found to vary between mice, and with different starting populations of sorted cells. This variation is not huge, and affects the size of subsets, rather than the representation amongst cells produced *in vitro*.

**Figure 3:**
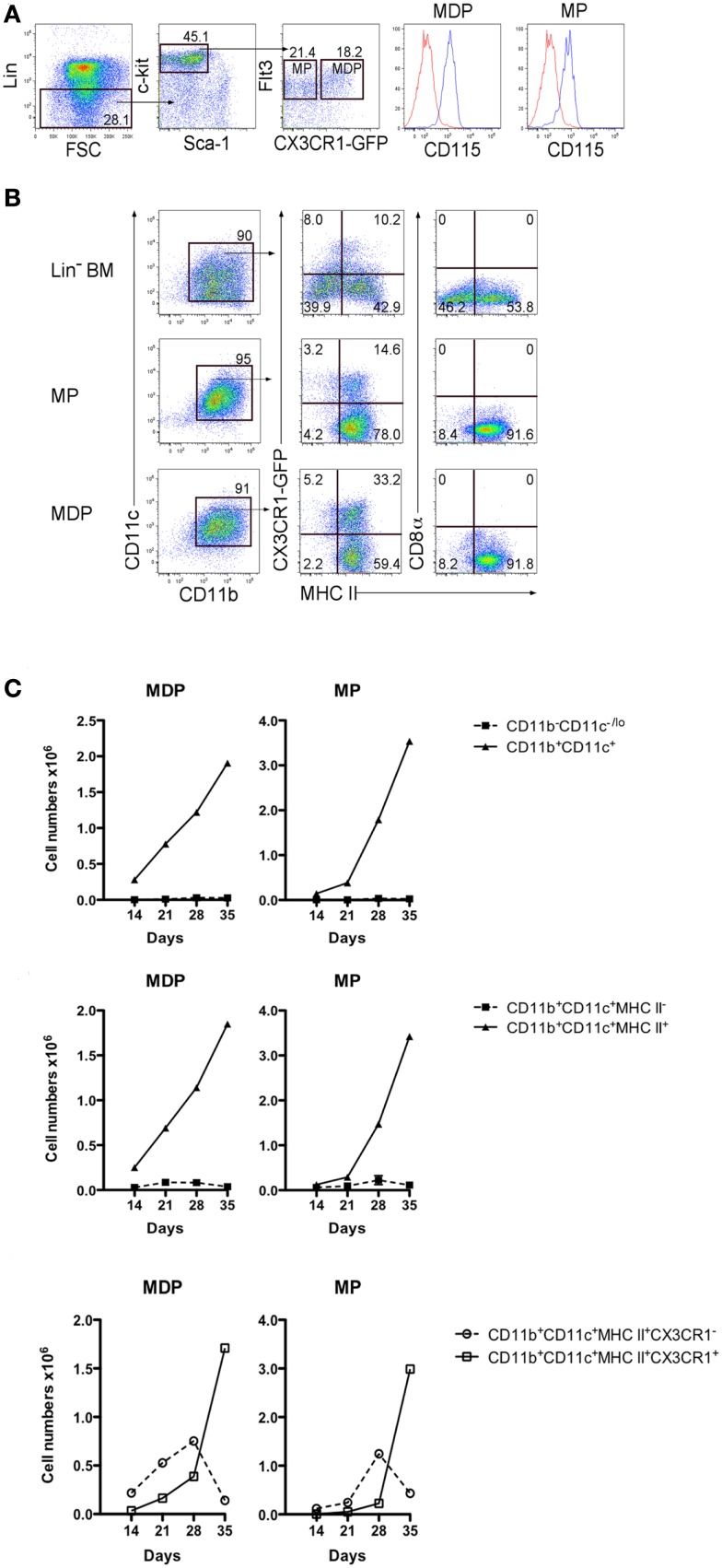
**MDP and MP are progenitors of only cDC-like cells in co-cultures**. **(A)** BM from CX3CR1-GFP mice was stained with antibodies and analyzed flow cytometrically for sorting MDP and MP as described in Figure 1. Lin^−^ cells were distinguished on a forward scatter (FSC) plot and c-kit^hi^ cells gated to distinguish Flt3^+^ cells. These were then sorted as CX3CR1^+^ and CX3CR1^−^ subsets of MP and MDP, respectively, each of which showed CD115 staining. **(B)** Sorted cells were co-cultured over 5G3 stroma using Lin^−^ BM as a control. Non-adherent cells were collected from co-cultures at different time points and stained for CD11c, CD11b, MHC-II, and CD8α to detect production of CD11b^+^CD11c^+^CD8α^−^B220^−^MHC-II^−^ L-DC and CD11b^+^CD11c^+^CD8α^−^B220^−^MHC-II^+^ cDC-like cells, and their expression of CX3CR1. Data shown reflect cells produced at 21 days. **(C)** Cell production was calculated in terms of number of cells of each type produced. Graphs show mean ± SE for triplicate co-cultures.

As a further test of progenitor potential, MDP were sorted on the basis of their expression of CX3CR1, which delineates them from described MP (14, 15). Lin^−^ BM from CX3CR1-GFP mice was stained to isolate c-kit^hi^Flt3^+^CD115^+^ cells differing in expression of CX3CR1, so reflecting the MDP and MP subsets (Figure 3A). These subsets were compared with Lin^−^BM from CX3CR1-GFP mice for capacity to seed 5G3 co-cultures (Figure 3B). Non-adherent cells were collected and analyzed flow cytometrically at 14, 21, 28, and 35 days to delineate L-DC and cDC-like cell production. Only MHC-II^+^ cDC-like cells were produced by MDP and MP co-cultures, with subsets of cDC-like cells expressing CX3CR1 evident at 21 days (Figure 3C). By contrast, Lin^−^ BM produced an extra subset of MHC-II^−^ L-DC, the majority of which were CX3CR1^−^ cells (Figure 3B). Co-cultures of MP produced an overall twofold increase in production of cDC-like cells over MDP co-cultures (Figure 3C). MDP and MP co-cultures did not maintain CD11b^−^CD11c^−/lo^ progenitors, nor support production of CD11b^+^CD11c^+^MHC-II^−^L-DC (Figure 3C). Furthermore, the production of CX3CR1^+^ cDC-like cells over similar CX3CR1^−^ cells increased over 35 days of co-cultures (Figure 3C).

**Figure 4:**
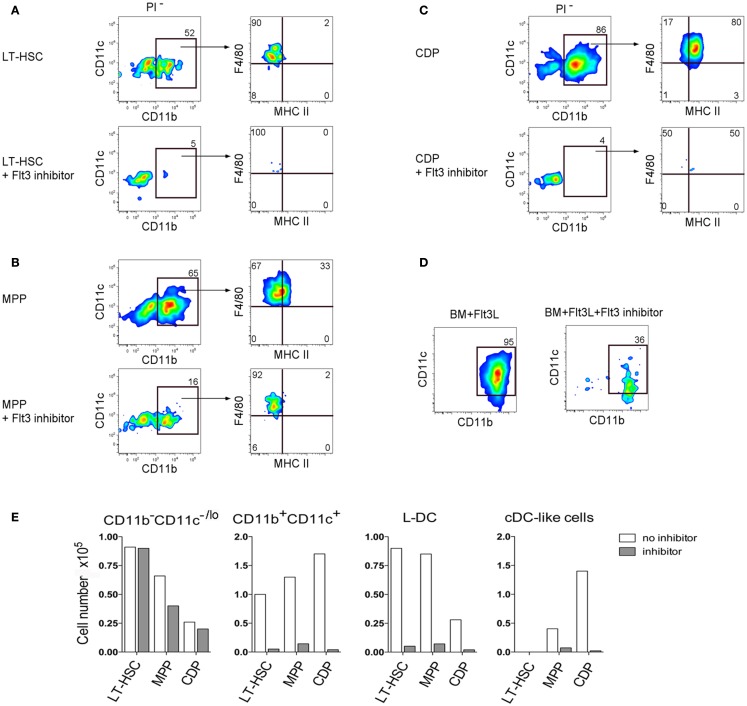
**Importance of Flt3 signaling for hematopoiesis in co-cultures**. **(A)** LT-HSC, **(B)** MPP, and **(C)** CDP were sorted from BM of C57BL/6J mice as in Figure 1, and established in co-cultures above 5G3 stroma in the presence and absence of Flt3 inhibitor. After 28 days cell production was assessed by staining with antibodies to CD11c, CD11b, F4/80, and MHC-II and flow cytometric gating of CD11c^+^CD11b^+^ cells and their MHC-II^−^ (L-DC) and MHC-II^+^ (cDC-like cell) subsets. **(D)** Control BM cultures were established with Flt3L in the presence and absence of the Flt3 inhibitor, and production of CD11c^+^CD11b^+^ cells was determined after 7 days. **(E)** Production of different cell types for each of the co-cultures is shown in terms of absolute number of cells produced.

One explanation for the presence of L-DC progenitors amongst both the LT-HSC and MPP subsets is that the LT-HSC progenitor undergoes differentiation and expression of Flt3 in co-cultures, such that two closely related L-DC progenitors exist in BM differing in expression of Flt3. This hypothesis was proven correct when a Flt3-specific inhibitor was added into co-cultures of LT-HSC and MPP which are Flt3^−^ and Flt3^+^ progenitors, respectively. This inhibitor almost completely blocked the formation of CD11b^+^CD11c^+^MHC-II^−^F4/80^+^ L-DC-like cells after 28 days of co-culture, with cultures still retaining a high proportion of CD11b^−^CD11c^−/lo^ cells containing progenitors (Figures 4A,B). This inhibitor also completely blocked development of cDC-like cells in 28-day co-cultures established with sorted Flt3^+^ CDP, and left the CD11b^−^CD11c^−/lo^ progenitor-containing subset intact (Figure 4C). In control Flt3-induced BM cultures, cDC production was lost by 7 days (Figure 4D). This experiment was analyzed at 28 days to allow more time to see the effect of inhibitors. However 28 days is the limit of co-culture reliability, and the absence of CD11c upregulation between CD11b^−^ and CD11b^+^ cells is probably due to the downturn in cell development.

While others have reported pDC production in these cultures (22), we have been unable to reproduce this finding using staining for CD11c, CD11b, MHC-II, and B220 (all data not shown) (Figure 4D). The Flt3 inhibitor was not toxic to cultured cells since there was no change in 5G3 stroma only cultures (data not shown). The best evidence of non-toxicity of the Flt3 inhibitor in these experiments is that the progenitor-containing CD11b^−^CD11c^−/lo^ subset was maintained at near control numbers (Figure 4E). These data confirm the importance of Flt3 signaling in L-DC development from progenitors, as well as in the development of cDC, although the latter arise from a distinct CDP which does not give rise to L-DC.

### Further definition of L-DC progenitors

**Figure 5:**
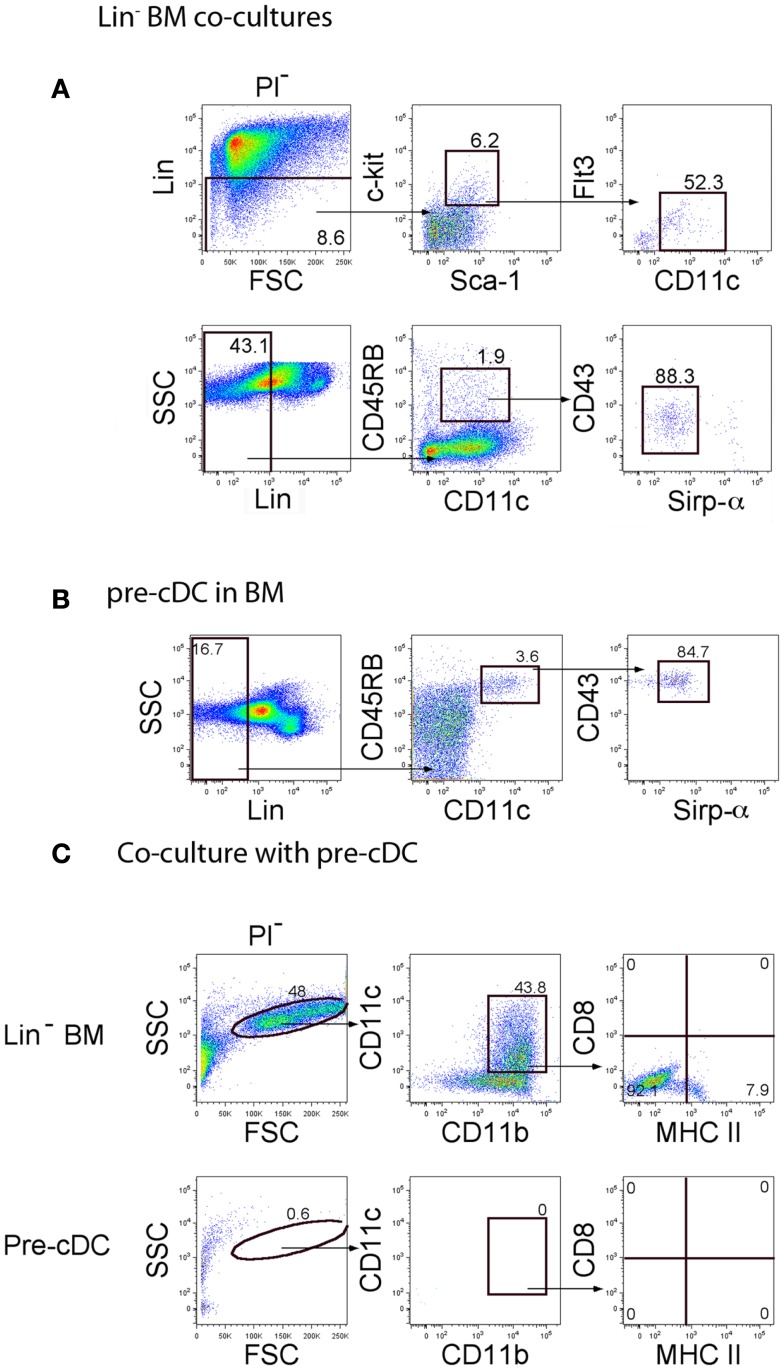
**Investigation of pre-cDC production in co-cultures**. The presence of pre-cDC was investigated in **(A)** cells collected from 14-day co-cultures of C57BL/6J Lin^−^ BM over 5G3 stroma, and in **(B)** C57BL/6J BM. Progenitors were gated as a Lin^−^ subset using FSC and SSC plots. Pre-cDC were detected by staining for c-kit, Sca-1, Flt3, CD11c, CD45RB, CD43, and Sirpα. Propidium iodide (PI) was added to allow gating of PI^−^ live cells. Gates were set on bivariate plots using isotype control antibodies, and numbers on gates reflect % positive cells. Using the gating strategy shown in **(B)**, pre-cDC were sorted out of BM for co-culture above 5G3 stroma. **(C)** Production of dendritic-like cells in pre-cDC co-cultures was assessed in relation to Lin^−^ BM co-cultures after 14 days following gating of large cells on a bivariate CD11c versus CD11b plot, and analysis of expression of CD11c, CD11b, MHC-II, and CD8α.

Further experiments addressed the question of whether pre-cDC could seed co-cultures for hematopoiesis since co-cultures of Lin^−^ BM over 5G3 were found to produce Lin^−^CD45RB^+^CD43^lo^Sirpα^lo^ cells described previously as pre-cDC (22, 23) (Figure 5A). The presence of a Lin^−^c-kit^+^Sca1^+^CD11c^lo^Flt3^−^ subset was also considered indicative of DC precursors. When Lin^−^CD45RB^+^CD43^lo^Sirpα^lo^ pre-cDC were sorted out of BM (Figure 5B) and overlaid on 5G3 in co-cultures, they were however unable to produce either cDC-like or L-DC-like cells after 14 days, a result which emphasizes their more advanced differentiative state (Figure 5C).

**Figure 6:**
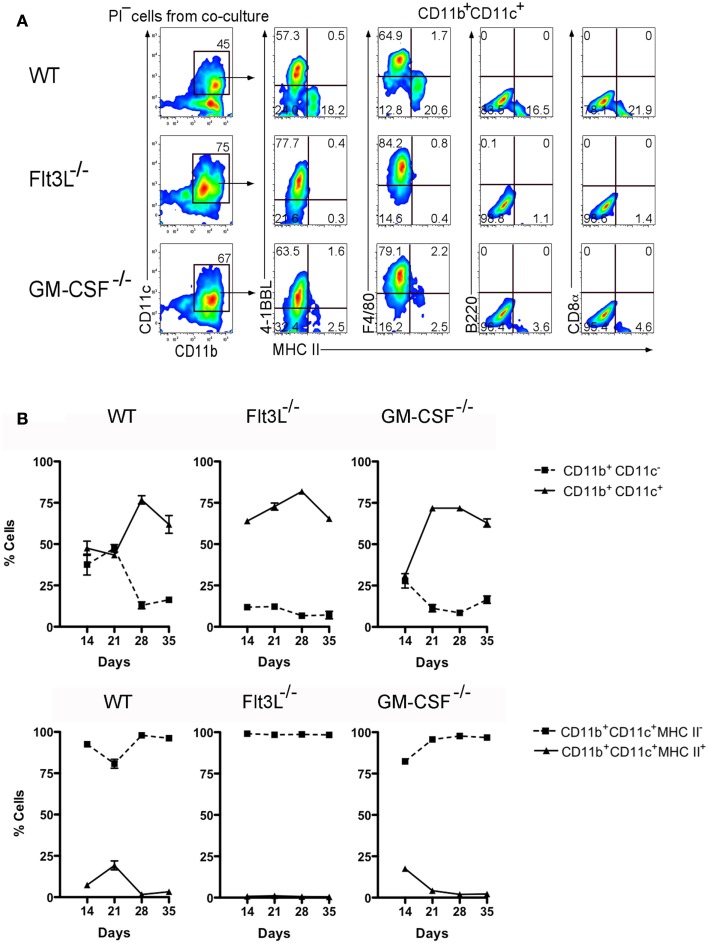
**BM from Flt3L^−/−^ and GM-CSF^−/−^ mice contains progenitors of L-DC but not cDC-like cells**. Lin^−^ BM prepared from C57BL/6J (WT), Flt3L^−/−^, and GM-CSF^−/−^ mice, was overlaid on 5G3 stroma and cell production monitored weekly for 5 weeks. **(A)** Non-adherent cells were stained with antibody to detect a CD11b^+^CD11c^+^ population of dendritic-like cells, then tested for expression of MHC-II, 4-1BBL, F4/80, CD8α, and B220. Data shows cell production at 21 days for one of three replicate cultures established from different mice. Gates were set on bivariate plots using isotype control antibodies and numbers on gates reflect % positive cells. **(B)** Co-cultures were analyzed over time for production of CD11b^+^ cells differing in expression of CD11c, and for production of CD11c^+^CD11b^+^ cells differing in expression of MHC-II. Proportion of each subset is shown as mean ± SE for triplicate co-cultures. All data are significantly different (*p* ≤ 0.05), with several exceptions: WT on days 14 and 21, and GM-CSF^−/−^ on day 14.

In order to further distinguish the functional potential of L-DC in relation to cDC-like cells, Lin^−^ BM from Flt3L^−/−^ and GM-CSF^−/−^ mice, and from syngeneic control C57BL/6J wild-type (WT) mice were tested for differentiative capacity in 5G3 co-cultures. These mice have very reduced numbers of cDC and pDC and significantly fewer progenitors of cDC and pDC in BM (24). Subset analysis of spleen has revealed that the L-DC subset is present in both of these strains (unpublished data). Cell production was monitored flow cytometrically by staining cells collected from triplicate co-cultures for CD11b, CD11c, MHC-II, 4-1BBL, F4/80, B220, and CD8α (Figure 6A). CD11b^+^CD11c^+^ cells were gated for analysis of production of MHC-II^−^ L-DC and MHC-II^+^ cDC-like cells. In this experiment involving Lin^−^ BM overlays, the total yield of cells was equivalent across co-cultures limited only by the culture system. The production of cells is therefore only presented in terms of % cells of each subset. WT co-cultures at 21 days showed a majority (~60%) population of MHC-II^−^ L-DC distinguishable from a smaller population (~20%) of MHC-II^+^ cDC-like cells, by F4/80 and 4-1BBL expression (Figure 6A). Expression of 4-1BBL is also consistent with the capability of L-DC to induce CD8^+^ T cell activation and proliferation (25). Again, only the two types of cells were produced with no evidence of B220^+^ pDC or CD8α^+^ cDC. A comparison of mutant and WT co-cultures also revealed loss of CD11b^+^CD11c^−^ myeloid precursors in BM of Flt3L^−/−^ and GM-CSF^−/−^ mice. This was evident in terms of reduction in the proportion of myeloid versus dendritic-like cells produced over time (Figure 6B). The DC population arising from progenitors in Flt3L^−/−^ and GM-CSF^−/−^ BM cultured over 5G3 stroma was exclusively L-DC (Figure 6B). This study served to distinguish the progenitors of cDC from those of L-DC, and to further distinguish L-DC as F4/80^+^4-1BBL^+^ cells. The reproducibility of results is again represented by the smaller error values achieved by staining cells produced in triplicate co-cultures. These findings are consistent with former reports showing a shortage of cDC precursors in mice harboring Flt3 and Flt3L mutations (24, 26). A smaller reduction in production of cDC-like cells in GM-CSF^−/−^ co-cultures supported evidence that GM-CSF is dispensable for DC development (24).

### M-CSF produced by stroma is essential for development of only cDC-like cells

**Figure 7:**
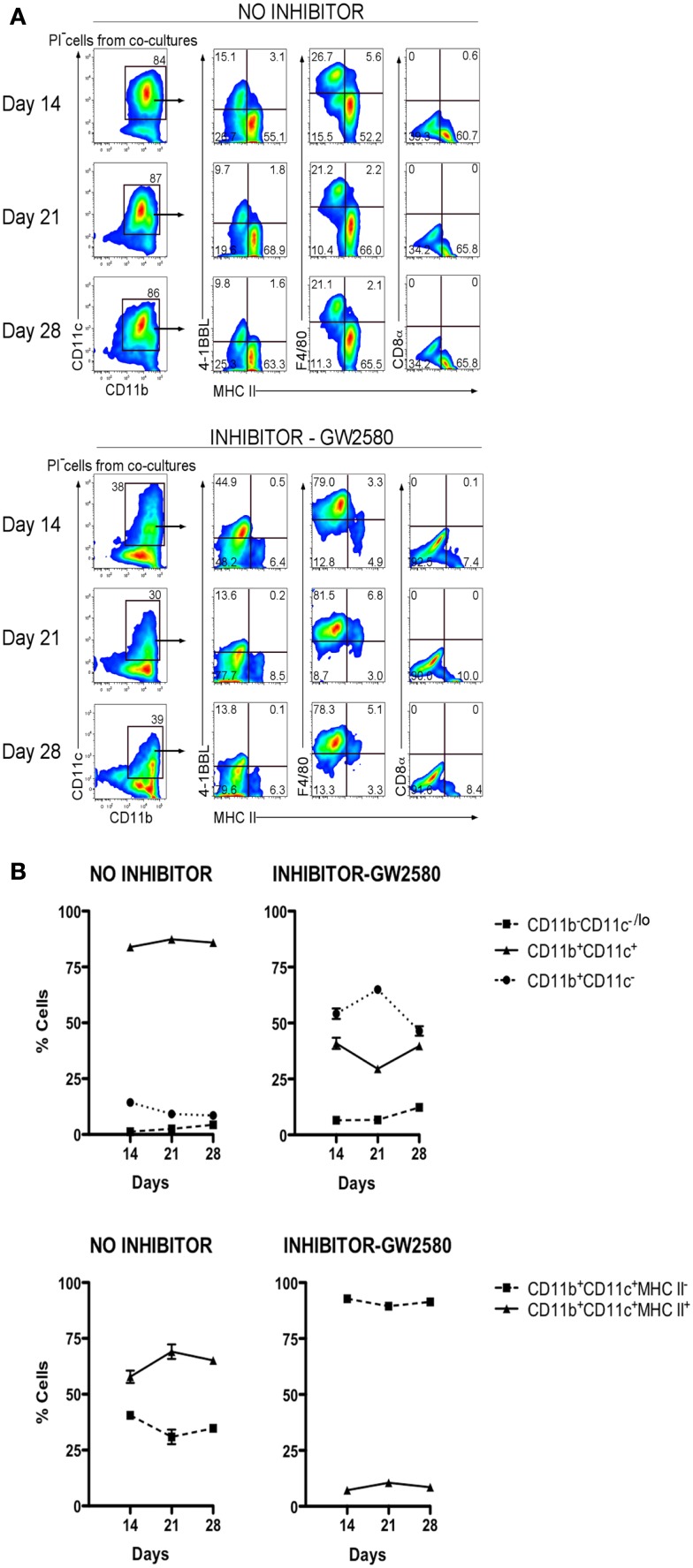
**M-CSFR inhibitor selectively inhibits development of cDC-like cells but not L-DC**. Lin^−^ BM from C57BL/6J mice was co-cultured with 5G3 stroma in the absence or presence of 10 nM GW2580, an inhibitor of M-CSFR, added again to cultures at medium change every 3–4 days. **(A)** Cell production was analyzed at 14, 21, and 28 days, by staining cells for CD11c, CD11b, MHC-II, 4-1BBL, F4/80, and CD8α, followed by flow cytometric analysis. Propidium iodide staining was used to delineate live (PI^−^) cells, which were then gated on CD11c and CD11b using isotype controls to set gates. L-DC were then distinguished from cDC-like cells on the basis of expression of 4-1BBL, F4/80, and the absence of MHC-II expression. **(B)** Co-cultures were analyzed over time for production of CD11b^+^ cells differing in expression of CD11c, and for production of CD11c^+^CD11b^+^ cells differing in expression of MHC-II. Proportion of each subset is shown as mean ± SE for triplicate co-cultures. All data are significantly different (*p* ≤ 0.05).

Previously M-CSF has been shown to support the development of cDC and pDC *in vitro*, and to increase the production of these cells *in vivo*, particularly in Flt3L^−/−^ mice which have very reduced numbers of mature cDC and pDC (27). The functional importance of M-CSF produced by 5G3 stroma (28) was therefore tested by inclusion of the GW2580 inhibitor of M-CSFR (29) in co-cultures established with Lin^−^ BM. Inhibitor was maintained in co-cultures out to 28 days. The production of cells was monitored and found to be equivalent across all co-cultures as common in Lin^−^ BM co-cultures. The proportion of L-DC and cDC-like cells produced was monitored flow cytometrically and the data presented as % cells of each type in terms of mean ± SE of triplicates. In the absence of GW2580, these co-cultures produced a large subset of CD11b^+^CD11c^+^MHC-II^+^ cDC-like cells. The 50–60% subset of cDC-like cells maintained for 28 days was however reduced to a 6–8% population in co-cultures containing GW2580 (Figure 7A). This particular experiment was selected out of several replicates for presentation based on its higher production of cDC-like cells compared with L-DC. Differences in the proportion of these cell types can occur and appear to reflect age-related and individual animal effects. Co-cultures treated with inhibitor showed an increase in the proportion of CD11b^+^CD11c^−^ myeloid precursors, with a concomitant reduction in the proportion of CD11b^+^CD11c^+^ cells (Figure 7B). The progenitor-containing subset of CD11b^−^CD11c^−/lo^ was consistently low. L-DC produced in co-cultures with added inhibitor were detectable on the basis of their unique expression of 4-1BBL, F4/80, and absence of MHC-II. The relative proportion of these cells increased to near 100% in co-cultures containing inhibitor. While cDC-like cells produced in 5G3 stromal co-cultures are dependent on M-CSF for development, L-DC are clearly not.

## Discussion

This study focuses on the developmental origin of two functionally distinct dendritic-like cell types produced *in vitro* over splenic stroma. The 5G3 stromal line as a supporter of hematopoiesis is unique and under independent investigation in relation to stromal elements which form microenvironments for hematopoiesis in BM. A single main cell type namely “L-DC” is produced in co-cultures of BM over 5G3 stroma. L-DC are novel as MHC-II^−^ DC with high capacity for endocytosis of antigen and cross-presentation with activation and proliferation of CD8^+^ T cells but not CD4^+^ T cells (5, 8, 9). Co-cultures of mixed BM however also show transient production of cells reflective of cDC (6–8). These findings raise the possibility that spleen may play a role in myelopoiesis in addition to its dominant role in erythropoiesis. This possibility is made more likely due to evidence that spleen contains a phenotypically and functionally similar “L-DC” subset with similar high endocytic capacity and capacity for strong activation of CD8^+^ T cells but no or weak activation of CD4^+^ T cells (9). It is this latter property of cross-presentation, along with cell morphology and CD11c expression which has led to classification of this cell as dendritic-like rather than myeloid or macrophage-like. L-DC do not express markers of splenic macrophages like MOMA-1, CD68, and SIGNR1, although they more closely resemble residential and inflammatory monocytes. Their relationship with inflammatory or residential macrophages, which do not cross-present antigen, is under further investigation. The important role of distinct antigen presenting cells in a variety of immune responses justifies our further characterization of the L-DC subset.

The progenitors of both L-DC and cDC-like cells produced in co-cultures have been investigated here with a view to determining their lineage origin in relation to other previously defined dendritic and myeloid cell types. Co-cultures initiated with known HSPC subsets sorted from BM show differential capacity to produce the distinct L-DC and cDC-like subsets which are produced together in co-cultures established with a heterogeneous Lin^−^ BM subset (7, 10). Both MDP, as progenitors of monocytes and DC, as well as CDP, the more restricted progenitor of cDC and pDC, both produce only cDC-like cells in co-cultures. This distinguishes L-DC immediately from the more common DC lineage comprising CD8^+^ cDC, CD8^−^ cDC, and pDC subsets which constitute the largest subsets of DC present in spleen. MDP were also isolated along with the earlier MP on the basis of differential expression of CX3CR1, and these progenitor subsets both produced only cDC-like cells, with no evident maintenance of progenitors within co-cultures. All of this evidence serves to distinguish L-DC as a cell type distinct from the common myeloid and DC lineages. The likeness of co-culture derived cDC-like cells to commonly described cDC is based on phenotypic and functional similarity, i.e., capacity of those cells to activate CD8^+^ and CD4^+^ T cells (10). Over many repeat experiments we have never recorded the production of CD8^+^ cDC or of B220^+^ pDC in 5G3 stromal co-cultures.

Long-term hematopoietic stem cells, isolated out of BM as a CD150^+^Flt3^−^c-kit^+^Lin^−^Sca-1^+^ subset (19), yielded only L-DC-like cells in co-cultures along with a high frequency of progenitors. Stroma appears to support restricted and direct differentiation of LT-HSC to L-DC. The L-DC progenitor appears to be maintained in co-cultures for extended periods of 5 weeks shown here, and up to 6 months (data not shown). The MPP, which is a progenitor subset immediately downstream of LT-HSC, also produces predominantly L-DC-like cells in co-cultures. Either the same progenitor must be present in both subsets which differ by expression of Flt3 and CD150, or two distinct progenitors must exist, one being the derivative of the other. This finding is consistent with a study showing that all hematopoietic lineages develop through a Flt3^+^ intermediate, the expression of which demarcates the loss of self-renewal capacity amongst HSC (30). Indeed by using a Flt3 inhibitor we demonstrated the functional importance of Flt3 signaling in the development of L-DC such that the Flt3^−^ LT-HSC subset must have acquired Flt3 expression during co-culture. A role for Flt3L in L-DC development is predicted, but is not supported by evidence for high production of Flt3L by 5G3 (data in preparation). The production of Flt3L by 5G3, as well as the possibility of alternate ligands for Flt3, are under further consideration. Further experiments have also distinguished the developmental origin of L-DC by lack of progenitor expression of CD115, and lack of dependency on M-CSF.

L-DC progenitors in BM were investigated in relation to the known CDP, MDP, MP, and HSC progenitors described previously. However our previous studies have also partially characterized the L-DC progenitor in spleen as a Lin^−^c-kit^lo^ subset reflecting HSC (11, 13, 31). Indeed, spleen does contain low numbers of HSC which can fully reconstitute the hematopoietic system of irradiated mice and also give rise to L-DC (7, 11, 32). By comparison with the *in vitro* co-culture studies described here, the *in vivo* reconstitution studies revealed multilineage development and no restricted differentiation of L-DC (30, 32). However, those studies did show clear evidence of a predominance of L-DC amongst all DC produced in spleen, suggesting that the splenic microenvironment may be more conducive to L-DC development (30, 31, 33). The possibility that L-DC reflect an antigen presenting cell type endogenous to spleen is therefore under further investigation.

Overall, this study demonstrates that the progenitor of L-DC is distinct from the common progenitor of DC (CDP), monocytes/DC (MDP), and myeloid cells (MP) present in BM. In 5G3 co-cultures, L-DC-like cells can be differentiated directly from the earliest self-renewing HSC, the LT-HSC, while cDC-like cells require MP, MDP, or CDP to maintain their numbers within co-cultures. L-DC development is also functionally distinguished since it occurs independently of M-CSF and GM-CSF, while these factors influence the development of cDC-like cells in co-cultures and in normal development in the animal. While *in vitro* studies may not exactly reflect *in vivo* development, it is notable that the well described *in vivo* subsets of cDC and pDC can be generated in cultures dependent on Flt3L (22), and monocyte-derived DC can be generated *in vitro* in the presence of GM-CSF (34). M-CSF-dependent cultures have also been shown to generate cells resembling cDC and pDC (27). Since the *in vivo* equivalent of L-DC has been partially characterized in murine spleen (9), the production of similar cells *in vitro* does not appear artifactual.

## Author Contributions

Sawang Petvises: performance of experiments, analysis and assembly of data, manuscript writing. Helen Christine O’Neill: design of the project, interpretation and analysis of data, manuscript writing.

## Conflict of Interest Statement

The authors declare that the research was conducted in the absence of any commercial or financial relationships that could be construed as a potential conflict of interest.
